# Multi-Omics Analysis Reveals Age-Related Enhancements in Gut Morphology, Microbiome, and Metabolism of Tibetan Pigs

**DOI:** 10.3390/microorganisms14051162

**Published:** 2026-05-21

**Authors:** Mengshi Zhao, Hongyang Zhao, Peimin Li, Song Peng, Fengqiang Lin, Quanwang Wu, Phurbu Tashi, Zhaolong Li

**Affiliations:** 1 Gut Microbiome Research Center, Institute of Animal Husbandry and Veterinary Medicine, Fujian Academy of Agricultural Sciences, Fuzhou 350013, China; 13375001253@163.com (M.Z.);; 2 Changdu Animal Husbandry General Station, Changdu 854000, China

**Keywords:** Tibetan pig, gut microbiome, multi-omics, intestinal morphology, transcriptomics, host-microbe interaction, aging

## Abstract

Age-related changes in the gut significantly impact host health, yet the multi-omics dynamics during the maturation of Tibetan pigs remain unclear. This study aimed to investigate the morphological, microbial, metabolic, and transcriptomic transformations in the intestines of aging Tibetan pigs. We analyzed the ileum and colon of 1-year-old and 3-year-old Tibetan pigs using histological evaluation, 16S rRNA sequencing, metabolomics, and transcriptomics. Aging to 3 years significantly improved ileal architecture, notably increasing the villus height to crypt depth ratio. Older pigs exhibited higher colonic microbial diversity, a decreased *Firmicutes* to *Bacteroidota* ratio, and enrichment of homeostasis-associated taxa, including *Lactobacillus*, *Prevotellaceae*, and *Ruminococcaceae*. Metabolomics revealed higher abundance of certain metabolites, including docosahexaenoic and arachidonic acids, enriching lipid metabolism and bile secretion pathways. Transcriptomics identified 2363 differentially expressed genes in the ileum, primarily involved in immune regulation and nutrient digestion. Integrated analysis showed strong positive correlations between enriched microbes (*Lactobacillus porci*) and up-regulated host genes (*UGT2B31*, *CCL28*) governing intestinal homeostasis. The transition from 1 to 3 years of age in Tibetan pigs fosters a synergistic host-microbiome environment, enhancing intestinal barrier function, immune capacity, and metabolic efficiency.

## 1. Introduction

The Qinghai–Tibet Plateau, recognized as one of the highest and most extreme high-altitude regions globally, presents significant challenges to livestock survival due to its unique cold, hypoxic climate and seasonally fluctuating feed resources [1]. Tibetan pigs, a distinctive local breed in this region, have developed remarkable adaptive capabilities to the extreme conditions of the Qinghai–Tibet Plateau through long-term natural selection and semi-wild grazing domestication [2]. Unlike modern pig breeds raised under intensive conditions, the survival strategy of Tibetan pigs relies on the plasticity of their gut microecosystem and its finely tuned regulation of host metabolism [3]. This unique interaction between the environment and microbes not only maintains the energy balance of Tibetan pigs in extreme settings [4] but also shapes their specific gut microbial structure and metabolic characteristics. Under traditional semi-grazing conditions, age-related differences in the dietary structure of Tibetan pigs drive the succession of their gut microecosystem [5]. Juvenile Tibetan pigs (e.g., 1-year-olds) experience a period of rapid growth and development, primarily relying on artificial supplementation, which includes a relatively high proportion of crop concentrates in their diet and a stable nutrient supply. In contrast, adult Tibetan pigs are more deeply integrated into the natural environment. Their diet is diverse, encompassing high-fiber wild plants, tubers, roots, and agricultural by-products, which present more severe nutritional restrictions and challenges in fiber digestion. This dietary transition from feed-based to wild-foraging-based inevitably reshapes the compositional diversity of the gut microbiota, which in turn regulates host intestinal tissue morphology and metabolic homeostasis through microbial metabolite signaling, such as short-chain fatty acids [6]. Although existing studies have concentrated on the genetic adaptation of Tibetan pigs to the plateau environment, systematic analyses of how dietary transitions at different growth stages specifically regulate gut microbiota and transcriptomic changes, along with their association with the remodeling of intestinal structures, remain limited. In particular, comparative studies between the two key age stages of 1-year-old and 3-year-old pigs can elucidate how Tibetan pigs respond to increasing fiber loads and environmental stress by regulating the microbe-host interaction network. This paper aims to summarize the adaptive characteristics of Tibetan pigs in high-altitude environments, focusing on the specific effects of age-dependent dietary differences on gut microbiota, metabolites, transcriptomes, and intestinal tissue morphology. It seeks to provide new theoretical perspectives for elucidating the biological basis of Tibetan pigs’ roughage tolerance and cold/hypoxia resistance, while also offering scientific evidence for the protection and development of plateau-specific livestock resources. The selection of 1-year-old and 3-year-old pigs as the key study stages represents the transition from the rapid growth phase (juvenile) to the mature, stable adult phase, which is critical for understanding the long-term adaptation of Tibetan pigs to their environment.

## 2. Materials and Methods

### 2.1. Animal Experimental Design and Feeding Management

This study employed a single-factor experimental design, utilizing 40 Tibetan pigs aged 1 and 3 years, which were divided into two groups of 20 pigs each. The one-year-old Tibetan pigs were raised in a small-scale ecological system, primarily fed corn, wheat, and highland barley, with unrestricted access to food and water. In contrast, the three-year-old Tibetan pigs were managed under a semi-grazing system, with a diet predominantly comprising wild plants, tubers, roots, and agricultural by-products, while strictly adhering to epidemic prevention regulations. The full cohort of 40 pigs was used for longitudinal monitoring of growth performance, health status, and fecal sample collection; the remaining 34 animals were not euthanized and were kept for other ongoing studies. Ultimately, three pigs were randomly selected from each group (six in total) for slaughter and sampling due to the high cost of free-range Tibetan pigs and the destructive nature of tissue collection. No formal power analysis was conducted prior to the study, as this was an exploratory investigation. We acknowledge that the small sample size (*n* = 3 per group) limits the statistical power and generalizability of our findings. To mitigate this, we applied stringent statistical thresholds (VIP ≥ 1, |log_2_FC| ≥ 1, *p* ≤ 0.05). (Since the Tibetan pigs we collected were free-range and relatively expensive, the number of samples culled was limited. Future studies with larger cohorts are warranted to validate these findings and account for individual variability), which involved the collection of feces, intestinal contents, and intestinal tissue samples.

### 2.2. Sample Collection

Following the experiment, three pigs from each group (six in total) were randomly selected for slaughter. After dissection, the ileal and colonic contents were transferred to sterile 15 mL centrifuge tubes and stored at −80 °C for subsequent testing. Concurrently, ileal and colonic tissue samples were collected, gently rinsed with PBS to eliminate residual contents, and fixed in a 4% paraformaldehyde solution for the preparation of intestinal tissue sections.

### 2.3. Histomorphometric Analysis of the Intestine

Intestinal tissues were fixed in 4% paraformaldehyde, followed by routine dehydration, clearing, and paraffin embedding. Sections were cut at a thickness of 5 μm and stained with hematoxylin and eosin (HE) or Alcian blue-periodic acid-Schiff (AB-PAS). Three non-consecutive sections were prepared per sample, and five random fields of view per section were examined under a light microscope at ×40 magnification. The following parameters were measured: villus height (VH), crypt depth (CD), the villus height to crypt depth ratio (VH/CD), and goblet cell count (GCC). The CaseViewer 2.4.2119028 scanning and browsing software was used to select regions of interest within the tissue, and images were captured at the same magnification. During imaging, the tissue was positioned to fill the entire field of view as much as possible, ensuring consistent background illumination across all captured images. After imaging, Image-Pro Plus 6.0 analysis software was used for measurements, with millimeters set as the standard unit.

### 2.4. Intestinal Tissue Structure Morphology Measurement

The preparation process for hematoxylin and eosin (HE) sections primarily involves several steps: tissue collection and fixation, dehydration and clearing, paraffin embedding, sectioning and mounting, dewaxing and rehydration, HE staining, followed by dehydration and clearing, and final mounting. After completing these sequential processes, permanent sections suitable for microscopic observation are obtained. Similarly, the preparation of Alcian blue-periodic acid-Schiff (AB-PAS) sections includes section preparation, dewaxing to water, Alcian blue staining, periodic acid oxidation, Schiff reagent staining, hematoxylin staining, Scott’s bluing, and subsequent dehydration and mounting. This process also results in permanent sections suitable for microscopic examination. For each sample, three sections were prepared, and five fields of view were selected for each section to measure various parameters, including villus height (VH), villus surface area (VSA), crypt depth (CD), the villus height/crypt depth ratio (VH/CD), and goblet cell number (GCC). Data analysis was conducted using Caseviewer version 2.4.0.119028.

### 2.5. 16S rRNA Gene Amplicon Sequencing and Bioinformatics Analysis

Ileal and colonic content samples were collected separately, placed in sterile cryopreservation tubes, rapidly frozen in liquid nitrogen, and subsequently transferred to a −80 °C freezer for long-term storage. Total DNA was extracted using the QIAamp PowerFecal Pro DNA Kit (Qiagen, Hilden, Germany), and its integrity, purity, and concentration were assessed through 1% agarose gel electrophoresis and a Nanodrop spectrophotometer. The V3–V4 hypervariable region of the 16S rRNA gene was amplified using universal primers (338F: 5′-ACTCCTACGGGAGGCAGCA-3′ and 806R: 5′-GGACTACHVGGGTWTCTAAT-3′). Libraries were prepared using the NEBNext Ultra DNA Library Prep Kit (Ipswich, MA, USA)and sequenced on the Illumina NovaSeq 6000 platform (San Diego, CA, USA) with paired-end 250 bp reads (PE250). Raw data were filtered for quality using Trimmomatic (version 0.33), primer sequences were identified and removed using Cutadapt (version 1.9.1), and paired-end reads were assembled while removing chimeras (UCHIME, version 8.1) using USEARCH (version 10) to obtain high-quality sequences for subsequent analysis. Operational taxonomic units (OTUs) were clustered at 97% similarity using the UPARSE pipeline. Taxonomic assignment was performed by comparing representative sequences against the SILVA database (version 138) using a naive Bayesian classifier. Downstream analyses included alpha diversity (Chao1, Shannon, Simpson indices), beta diversity (Bray–Curtis dissimilarity and unweighted UniFrac), and linear discriminant analysis effect size (LEfSe) to identify differentially abundant taxa between groups.

### 2.6. Determination of Metabolites in Tibetan Pig Ileal and Colonic Contents

A total of 25 mg of samples was accurately weighed at low temperatures and placed into 1.5 mL EP tubes. To each tube, homogenization beads and 500 μL of an extraction solution, composed of methanol, acetonitrile, and water in a 2:2:1 (*v*/*v*) ratio, were added. This solution also contained isotope-labeled internal standards. The mixture was vortexed for 30 s, homogenized in a homogenizer at 35 Hz for 4 min, and subsequently ultrasonicated in an ice-water bath for 5 min. This ultrasonication step was repeated three times. Following this, the samples were stored at −40 °C for 1 h and then centrifuged at 4 °C at 12,000 rpm (with a centrifugal force of 13,800× *g* and a radius of 8.6 cm) for 15 min. The supernatant was then collected for analysis. All samples were combined in equal volumes of supernatant to create quality control (QC) samples for subsequent analysis, which were injected periodically throughout the run to monitor instrument stability and data reproducibility. For the analysis of polar metabolites, a Vanquish ultra-high-performance liquid chromatograph (Thermo Fisher Scientific, Waltham, MA, USA) was employed, utilizing a Waters ACQUITY UPLC BEH Amide column (Waters Corporation, Milford, MA, USA) (2.1 mm × 50 mm, 1.7 μm) for the separation of target compounds. Phase A consisted of an aqueous solution containing 25 mmol/L ammonium acetate and 25 mmol/L ammonia, while Phase B was composed of acetonitrile. The sample tray was maintained at a temperature of 4 °C, and the injection volume was set to 2 μL. Data acquisition for MS1 and MS2 was conducted using an Orbitrap Exploris 120 mass spectrometer (Thermo Fisher Scientific, Waltham, MA, USA), controlled by Xcalibur (version 4.4, Thermo Fisher Scientific, Waltham, MA, USA).

Untargeted metabolomics data were processed using Compound Discoverer 3.1 as described above (peak detection, alignment, normalization, and identification against mzCloud, mzVault, and MassList). Multivariate statistical analysis including principal component analysis (PCA) and orthogonal partial least squares discriminant analysis (OPLS-DA) were performed using SIMCA (v14.1). Differential metabolites were selected based on VIP ≥ 1, |log_2_FC| ≥ 1, and a false discovery rate (FDR) adjusted *p* ≤ 0.25 (Benjamini-Hochberg correction), as well as a nominal *p* ≤ 0.05 (Student’s *t*-test). The OPLS-DA model was validated by 200-fold permutation tests. Metabolic pathway enrichment analysis was carried out using MetaboAnalyst 5.0 with the KEGG database.

### 2.7. Transcriptome Sequencing of Tibetan Pig Ileal Tissue

Total RNA was extracted from ileal tissue samples of 1-year-old and 3-year-old Tibetan pigs (three animals per age group) using TRIzol reagent (Thermo Fisher Scientific, Waltham, MA, USA). RNA integrity and concentration were assessed with an Agilent 2100 Bioanalyzer (Agilent Technologies, Santa Clara, CA, USA), and high-quality RNA (RIN ≥ 7.0) was used for library preparation. mRNA was enriched using oligo (dT) magnetic beads, fragmented, and reverse-transcribed into cDNA. Libraries were constructed using the NEBNext Ultra RNA Library Prep Kit (New England Biolabs, Ipswich, MA, USA) and sequenced on the Illumina NovaSeq 6000 platform with paired-end 150 bp reads (PE150). Raw reads were trimmed with Trimmomatic (v0.33), aligned to the pig reference genome (Sscrofa11.1) using HISAT2 (v2.1.0), and gene counts were quantified with featureCounts (v1.6.3). Raw counts were normalized using FPKM for visualization purposes only. Differential expression analysis between the two age groups was performed using DESeq2 (v1.30.1) with a threshold of |log_2_FC| ≥1 and adjusted *p*-value (FDR) ≤ 0.05. Functional enrichment analyses (GO and KEGG) were carried out using clusterProfiler (v4.0).

### 2.8. Correlation Analysis

Spearman correlation analysis was performed to assess associations between gut microbiota and host gene expression. Microbial taxa included were those differentially abundant between age groups based on 16S rRNA sequencing (*p* < 0.05, |log_2_FC| ≥ 1). Host genes were selected from differentially expressed genes identified by DESeq2 (|log_2_FC| ≥ 2, padj < 0.05), and further filtered for functional relevance to intestinal barrier, immune regulation, nutrient metabolism, or detoxification based on GO/KEGG annotations. Spearman correlation coefficients and corresponding *p* values were calculated using the Lianchuan Bio platform. Results were visualized as a heatmap. No multiple testing correction was applied, as this analysis was exploratory.

### 2.9. Statistical Analysis

Experimental data and figures were processed and analyzed using GraphPad Prism 8.0 software and CaseViewer 2.4.2119028. A one-way ANOVA was conducted for inter-group comparisons, and significance levels were determined. Values of “*” indicate *p* < 0.05 (significant), “**” indicate *p* < 0.01 (highly significant), and “***” indicate *p* < 0.001 (extremely significant).

## 3. Results

### 3.1. Intestinal Morphology of 1-Year-Old and 3-Year-Old Tibetan Pigs

**Figure 1:**
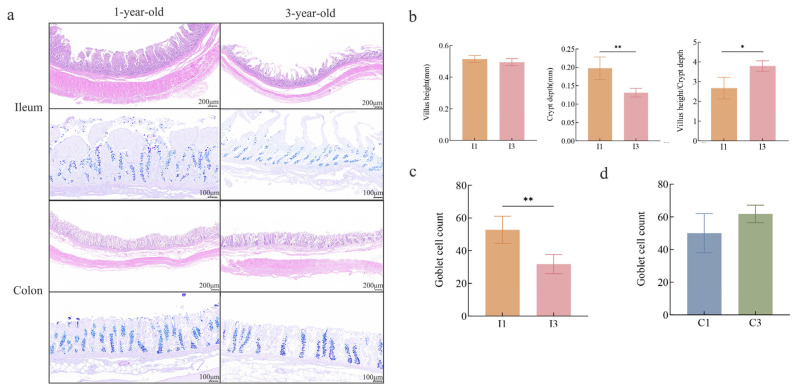
(**a**) Representative histological sections of the ileum and colon in 1-year-old (1Y) and 3-year-old (3Y) Tibetan pigs stained with HE and ABPAS. (**b**) Comparison of ileum villus height, crypt depth, and villus height/crypt depth ratio (V/C ratio) in Tibetan pigs between the 1Y and 3Y groups. (**c**) Quantification of goblet cell number in the ileum of 1Y and 3Y Tibetan pigs. (**d**) Quantification of goblet cell number in the colon of 1Y and 3Y Tibetan pigs. Data are presented as mean ± SEM (*n* = 3 per group). Statistical significance was determined by Student’s *t*-test; * *p* < 0.05, ** *p* < 0.01.

Hematoxylin and eosin (HE) staining (Figure 1a,b) showed that, compared to 1-year-old Tibetan pigs, ileal crypt depth in 3-year-old Tibetan pigs decreased significantly by 33.6% (*p* < 0.05), whereas villus height did not differ significantly between age groups. Consequently, the ratio of ileal villus height to crypt depth (V/C) in the ileum was 42% higher in 3-year-old pigs (*p* < 0.05). Alcian blue-periodic acid-Schiff (AB-PAS) staining (Figure 1a,c,d) revealed that ileal goblet cell number was significantly lower in 3-year-old than in 1-year-old Tibetan pigs. In contrast, colonic goblet cell number showed an increasing trend with age.

### 3.2. Microbial Diversity in the Ileum and Colon of 1-Year-Old and 3-Year-Old Pigs

**Figure 2:**
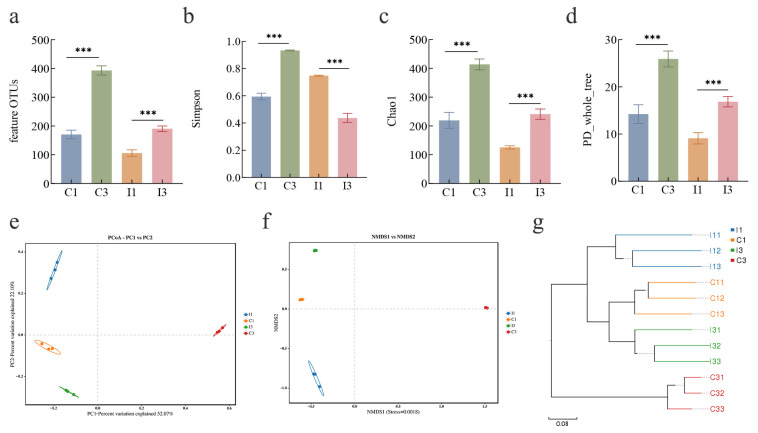
The analysis of Alpha diversity ((**a**) Feature _OUTs, (**b**) Simpson, (**c**) Chao1, (**d**) PD_whole_tree). The analysis of Beta diversity ((**e**) Principal Component Analysis, PCA; (**f**): Non-Metric Multi-Dimensional Scaling, NMDS; (**g**) Unweighted Pair-group Method with Arithmetic Mean). Statistical significance was determined by Student’s *t*-test; *** *p* < 0.001.

Tibetan Pigs Alpha diversity analysis demonstrated that the microbial richness and diversity in the colon of 3-year-old Tibetan pigs were higher than those in 1-year-old Tibetan pigs. In the ileum, the taxonomic richness of 3-year-old Tibetan pigs was greater than that of their 1-year-old counterparts, while community diversity was lower. Alpha diversity analysis utilized Chao1 and Ace indices to assess taxonomic richness, and Shannon and Simpson indices to evaluate community diversity. The results indicated that the Feature_OTUs, Chao1, Shannon, Simpson, and PD_whole_tree indices in the colon of 3-year-old Tibetan pigs were significantly higher than those in 1-year-old Tibetan pigs. In the ileum of 3-year-old Tibetan pigs, with the exception of the Shannon and Simpson indices, other indices were significantly elevated compared to those of 1-year-old Tibetan pigs (Table 1, Figure 2a–d). In summary, colonic microbial richness and community diversity were both higher in 3-year-old than in 1-year-old pigs. In the ileum, however, richness increased but diversity decreased with age.

### 3.3. Differences in Gut Microbiota of 1-Year-Old and 3-Year-Old Tibetan Pigs

**Figure 3:**
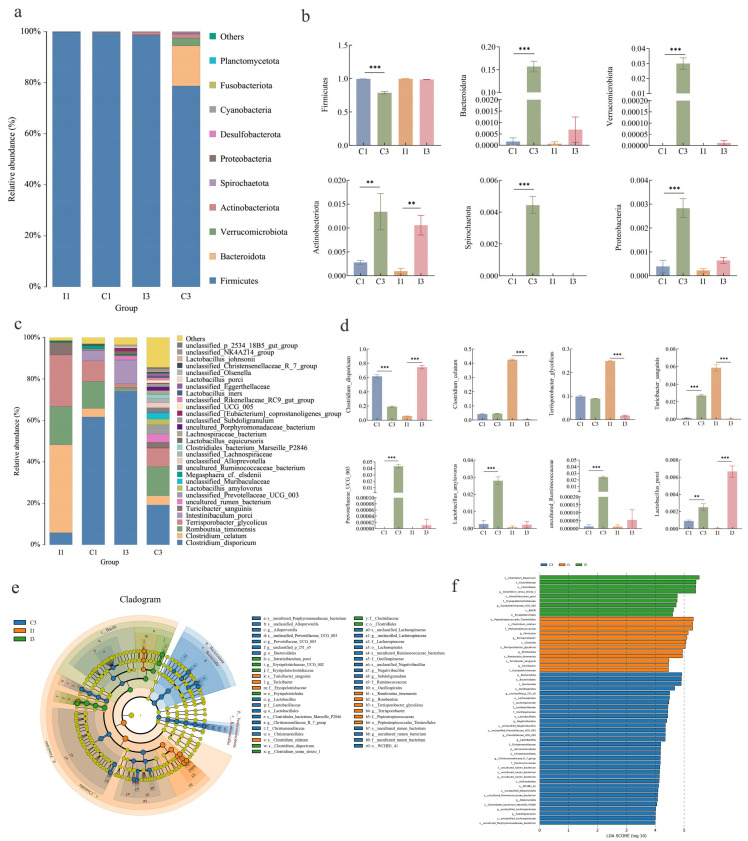
(**a**,**b**): Changes in intestinal microbiota composition at the phylum level ((**a**) stacked bar chart, (**b**) differentiated bacterial phylum). (**c**,**d**): Changes in intestinal microbiota composition at the genus level ((**c**) stacked bar chart, (**d**) differentiated bacterial species). (**e**,**f**): Analysis of LDA Effect Size ((**e**) Cladogram, (**f**) LDA score). Statistical significance was determined by Student’s *t*-test; ** *p* < 0.01, *** *p* < 0.001.

At the phylum level, the primary phyla identified between groups were *Firmicutes*, *Bacteroidota*, *Verrucomicrobiota*, *Actinobacteriota*, *Spirochaetota*, and *Proteobacteria*, with *Firmicutes* being the most dominant phylum, exhibiting a relative abundance exceeding 75% across all segments. Specifically, the abundance of *Firmicutes* in the colon of 3-year-old Tibetan pigs was significantly reduced (*p* < 0.01), while the abundances of *Bacteroidota* and *Verrucomicrobiota* were significantly increased (*p* < 0.01). Consequently, the ratio of *Firmicutes* to *Bacteroidota* was significantly decreased (Figure 3a,b).

At the genus and species level, we analyzed the top 30 abundant taxa (Figure 3c,d). Genera included *Prevotellaceae UCG 003*, uncultured *Ruminococcaceae*, *Megasphaera*, and *Olsenella*; species included *Clostridium disporicum*, *Clostridium celatum*, *Romboutsia timonensis*, *Terrisporobacter glycolicus*, *Lactobacillus porci*, and *Lactobacillus amylovorus*. The predominant species identified between groups included *Clostridium disporicum*, *Clostridium celatum*, *Romboutsia timonensis*, and *Terrisporobacter glycolicus*, all belonging to the class *Clostridia* within the phylum *Firmicutes*, and typically recognized as intestinal commensals. As illustrated in Figure 3c,d, the gut microbial diversity of 3-year-old Tibetan pigs significantly increased compared to their 1-year-old counterparts. In the colon, the abundance of *Clostridium disporicum* significantly decreased (*p* < 0.001), whereas the abundances of *Clostridium celatum* and *Romboutsia timonensis* showed no significant change. Conversely, the abundances of *Prevotellaceae UCG 003*, uncultured *Ruminococcaceae*, *Lactobacillus porci* and other taxa significantly increased (*p* < 0.001). In the ileum of 3-year-old Tibetan pigs, the abundances of commensal or opportunistic pathogens such as *Clostridium celatum*, *Romboutsia timonensis*, and *Terrisporobacter glycolicus* significantly decreased (*p* < 0.001), while other species such as *Clostridium disporicum* and *Lactobacillus porci* significantly increased (*p* < 0.001). Overall, the colon of 3-year-old Tibetan pigs showed increased abundance of several taxa including *Prevotellaceae*, *Ruminococcaceae*, *Lactobacillus*. LEfSe analysis revealed inter-group microbial differences. As depicted in Figure 3e,f, the microbial community exhibited significant changes. In the I1 group, differential features were predominantly concentrated in *o_Peptostreptococcales_Tissierellales*, *s_Clostridium_celatum*, *f_Peptostreptococcaceae*, and 12 other groups. In the I3 group, differential features were primarily concentrated in *s_Clostridium_disporicum*, *f_Clostridiaceae*, *o_Clostridiales*, and 9 other groups. In the C3 group, differential features were mainly concentrated in *P_Bacteroidota*, *o_Bacteroidales*, *c_Bacteroidia*, and 33 other groups, while the C1 group exhibited no differential features that were significantly distinct from other groups.

### 3.4. Differences in Gut Metabolites of 1-Year-Old and 3-Year-Old Tibetan Pigs

**Figure 4:**
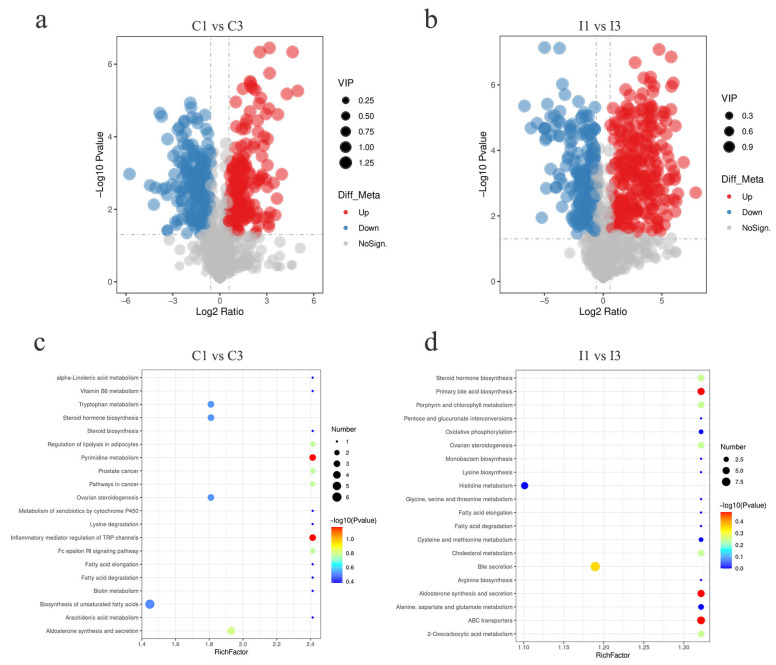
(**a**,**b**) Differential metabolite volcano plot. Red dots represent metabolites with higher abundance in the 3-year-old group, blue dots represent metabolites with lower abundance, and gray dots represent metabolites with no significant difference. (**c**,**d**) Differential metabolite enrichment analysis. Bubble chart showing significantly enriched metabolic and signaling pathways (*p* < 0.05), where bubble size represents the number of enriched genes and color represents the significance level.

Combining univariate and multivariate statistical analyses, differential metabolites were identified using Student’s *t*-test with a significance threshold of *p* < 0.05, a variable importance in projection (VIP) greater than 1 in the first principal component of the OPLS-DA model, and a fold change (FC) greater than 2 or less than 0.5. The statistical results indicated that there were 258 differential metabolites between the C1 and C3 groups, with 124 showing higher abundance and 134 showing lower abundance in the 3-year-old group. Notably, the significantly higher abundance metabolites included LPC O-17:1, docosahexaenoic acid, and arachidonic acid, whereas the significantly lower abundance included stercobilin, LPG 13:0, and histamine. Furthermore, there were 389 differential metabolites identified between the I1 and I3 groups, with 238 showing higher abundance and 150 showing lower abundance in the 3-year-old group. Metabolites with significantly higher abundance in this comparison included LPC O-20:2, 6-hydroxynicotinic acid, and ursolic acid, while metabolites with significantly lower abundance included D-pantethine, stercobilin, and L-threonic acid (Figure 4a,b). Based on analyses using the KEGG database, the related pathways of differentially expressed genes (DEGs) were examined. From the perspective of pathway enrichment analysis, the differential metabolites between the C1 and C3 samples were significantly enriched in pathways such as the biosynthesis of unsaturated fatty acids, tryptophan metabolism, and the inflammatory mediator regulation of TRP channels. In contrast, the differential metabolites between the I1 and I3 samples were significantly enriched in pathways such as histidine metabolism, bile secretion, and ABC transporters (Figure 4c,d).

### 3.5. Ileal Transcriptome of 1-Year-Old and 3-Year-Old Tibetan Pigs

**Figure 5:**
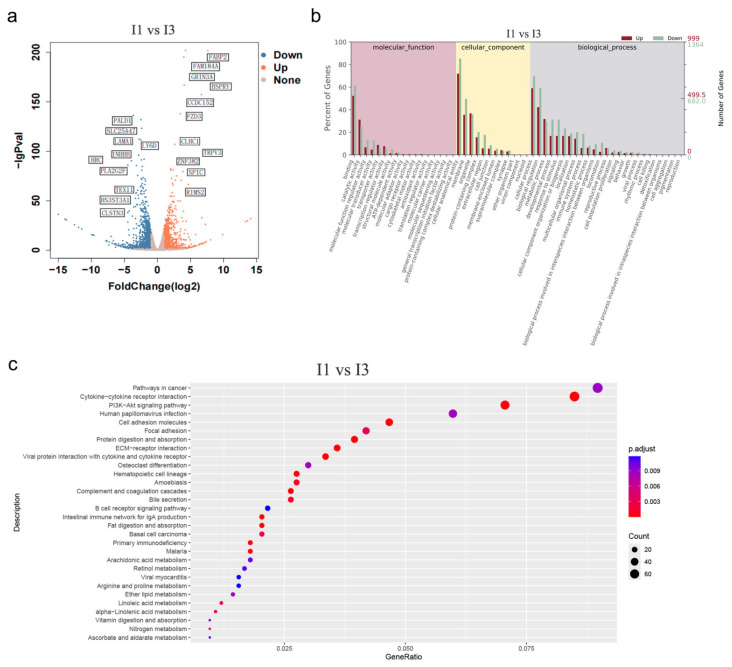
(**a**) Volcano plot of differentially expressed genes. Red dots represent significantly up-regulated genes, blue dots represent significantly down-regulated genes, and gray dots represent genes with no significant difference. (**b**) GO enrichment analysis. The enrichment results of differentially expressed genes in biological process (BP), cellular component (CC), and molecular function (MF) are shown. (**c**) KEGG pathway enrichment analysis of differentially expressed genes in ileal tissue. The bubble chart displays significantly enriched pathways (*p* < 0.05). The *y*-axis indicates pathway names, and the *x*-axis represents the Rich Factor. Bubble size indicates the number of differentially expressed genes enriched in each pathway, and bubble color represents the significance level.

Using DESeq2, we identified 2363 significantly differentially expressed genes between the two age groups (|log_2_FC| ≥ 2, *p* < 0.05, *q* < 0.05), including 999 up-regulated and 1364 down-regulated genes in the 3-year-old group (Figure 5a). Significant enrichment analysis was conducted via the GO database on the molecular function (MF), cellular component (CC), and biological process (BP) associated with the differentially expressed genes in the ileal tissue of Tibetan pigs (Figure 5b). GO enrichment analysis showed that differentially expressed genes were predominantly enriched in binding, catalytic activity, and molecular function regulator activity (molecular function); cellular anatomical entities, membranes, and organelles (cellular component); and cellular processes, biological regulation, and metabolic processes (biological process). Utilizing the KEGG database, we performed enrichment analysis on the differentially expressed genes in ileal tissue, identifying 30 significantly enriched pathways (*p* < 0.05). Notably, the significantly differentially expressed genes between I1 and I3 samples were enriched in pathways such as those associated with cancer, cytokine-cytokine receptor interaction, and the PI3K-Akt signaling pathway (Figure 5c). Among the enriched pathways, those related to immune regulation and nutrient metabolism were most prominently represented. Enriched pathways included immune-related pathways (the intestinal immune network for IgA production, cytokine-cytokine receptor interaction, and JAK-STAT signaling), nutrient metabolism-related pathways (protein, fat, and vitamin digestion and absorption, as well as bile secretion) lipid metabolism pathways (linoleic and arachidonic acid metabolism), amino acid metabolism pathways (arginine and proline metabolism), and xenobiotic metabolism pathways (cytochrome P450), in addition to barrier-related pathways (cell adhesion molecules and focal adhesion). These enrichment patterns suggest that the intestinal transcriptome of 3-year-old Tibetan pigs shifts toward enhanced immune regulation and metabolic flexibility, which may support adaptation to a fiber-rich plateau diet.

### 3.6. Correlation Analysis of Microorganisms and Differential Genes

**Figure 6:**
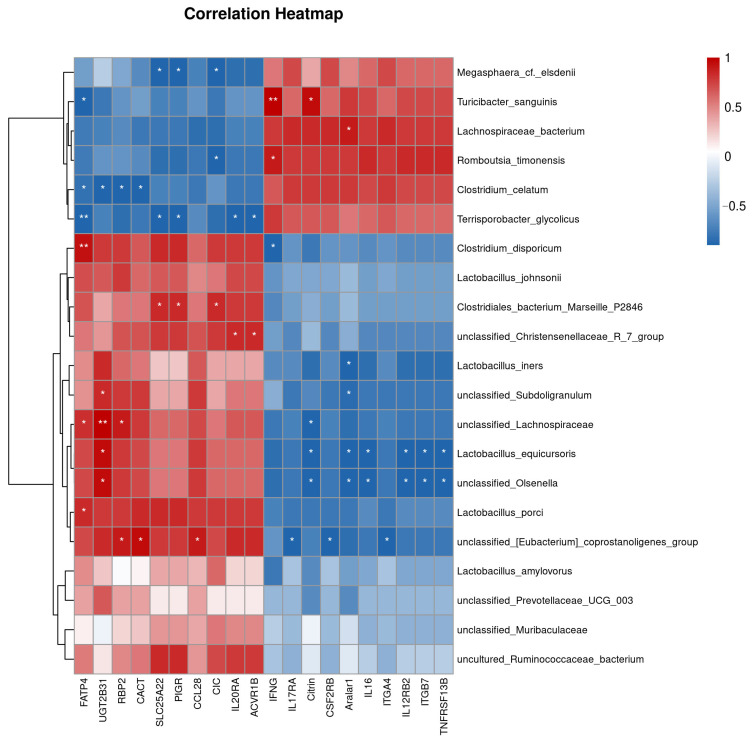
Correlation heatmap between gut microbiota and differentially expressed genes. Spearman correlation analysis was performed to assess associations between gut microbiota (at the species level) and differentially expressed genes. In the heatmap, each cell color represents the correlation coefficient, with red indicating positive correlation and blue indicating negative correlation; darker colors indicate stronger correlations. Asterisks denote significance levels (*: *p* < 0.05, **: *p* < 0.01). Differentially expressed genes are shown on the *x*-axis, and microbial taxa are shown on the *y*-axis.

Spearman correlation analysis was performed to examine associations between ileal microorganisms and differentially expressed genes in Tibetan pigs at different ages (Figure 6). The *UGT2B31* gene was significantly up-regulated in the ileum of 3-year-old Tibetan pigs compared to their 1-year-old counterparts. This gene is enriched in several metabolic pathways, including bile secretion, retinol metabolism, and cytochrome P450-mediated xenobiotic metabolism. Spearman correlation analysis detected a significant positive correlation between the expression level of *UGT2B31* and several bacterial genera, such as *Lactobacillus equicursoris*, unclassified *Lachnospiraceae*, unclassified *Subdoligranulum*, and unclassified *Olsenella*. Conversely, these microorganisms showed a significant negative correlation with most down-regulated differential genes. These correlational patterns suggest a potential association between *UGT2B31* and the host-microbiota interplay in intestinal homeostasis.

Additionally, the abundance of the [*Eubacterium*] coprostanoligenes group in the ileum of 3-year-old Tibetan pigs was significantly elevated and positively correlated with the expression levels of up-regulated differential genes, including *CCL28*, *RBP2*, and *CACT*. *CCL28* is known to be associated with immune and inflammation-related pathways, such as the intestinal immune network for IgA production, cytokine-cytokine receptor interaction, and viral protein interaction with cytokines and their receptors. *RBP2* has been linked to vitamin digestion and absorption, while *CACT* is primarily associated with fat digestion and absorption. Moreover, the abundance of *Lactobacillus porci* and *Clostridium disporicum* was significantly increased and positively correlated with the up-regulated differential gene *FATP4*.

## 4. Discussion

Tibetan pigs (TPs) exhibit remarkable adaptability to the harsh conditions of the Qinghai–Tibet Plateau, and the potential role of their gut microbiota warrants further investigation [7]. This study identified significant differences in intestinal tissue morphology, microbial abundance, metabolites, and gene expression between 1-year-old and 3-year-old Tibetan pigs. Compared to 1-year-old Tibetan pigs, the small intestinal crypt depth in 3-year-old Tibetan pigs was significantly reduced, while the number of goblet cells also decreased, and the villus-crypt ratio (V/C) significantly increased. In terms of gut microbiota, the richness and diversity of gut microorganisms in the colon of 3-year-old Tibetan pigs were significantly enhanced, and the bacterial community structure exhibited notable differences. Concurrently, metabolites with anti-inflammatory and antioxidant properties were significantly elevated; genes associated with energy metabolism and immune regulation were upregulated, indicating that the nutrient absorption efficiency and mucosal immunity of the intestines in 3-year-old Tibetan pigs were improved, contributing to the stability of their body tissues.

From a biological perspective, these changes suggest an age-related shift in intestinal resource allocation: reduced investment in a physical barrier (fewer goblet cells and shallower crypts) is offset by enhanced microbial diversity and metabolic flexibility, optimizing energy extraction from a fiber-rich plateau diet. Adaptive changes in its morphological structure represent key strategies for Tibetan pigs to cope with extreme environments [8]. This study revealed that as Tibetan pigs matured from 1 to 3 years of age, significant alterations in intestinal morphology occurred. The crypt depth decreased significantly by 33.6%. Despite the stability of villus height, these changes collectively led to a significant 42% increase in the villus-crypt ratio (V/C). This shift suggests that as Tibetan pigs age, their intestinal function transitions from maintaining a robust physical barrier to enhancing nutrient absorption. The colon exhibited an increasing trend in the number of goblet cells in 3-year-old Tibetan pigs, which may strengthen mucus secretion and facilitate the management of a more complex microbial ecosystem.

Previous studies have indicated that the gut microbiota of Tibetan pigs contributes to their survival on the Qinghai–Tibet Plateau [9]. Research indicates that the digestive tract microbiota of Tibetan pigs enhances their survival through specific microbial functions. Notably, their gut microbiota exhibits significant compositional and functional differences compared to that of pure black pigs, with key microorganisms such as *Bifidobacterium* and *Megasphaera* being notably enriched. These microbial groups possess the ability to degrade complex carbon sources and produce short-chain fatty acids, thereby facilitating efficient energy metabolism under hypoxic conditions [5]. This study revealed that alpha diversity (including OTUs, Chao1, Shannon, Simpson, and PD_whole_tree indices) in the colon of 3-year-old Tibetan pigs was significantly higher than in 1-year-old Tibetan pigs. Enhanced microbial diversity is generally regarded as an indicator of a more stable and healthy gut microecosystem. The clinical implications for livestock production are noteworthy, the identified age-specific microbial signatures, such as the increased abundance of *Lactobacillus amylovorus* and butyrate-producing *Clostridium* species, could serve as targets for probiotic development. Administering these strains to young piglets may accelerate gut maturation, reduce post-weaning diarrhea, and decrease reliance on antibiotic growth promoters, thereby supporting sustainable pig farming practices.

The *Firmicutes* phylum is abundant in genes associated with polysaccharide fermentation, which facilitates the conversion of dietary fiber into short-chain fatty acids (SCFAs), providing additional energy for the host. Studies have demonstrated that *Firmicutes* and *Bacteroidota* are predominant in the feces of Tibetan pigs, and their prevalence on the Qinghai–Tibet Plateau may hold significant adaptive importance [10]. In this study, Firmicutes was the dominant phylum (>75% abundance) in both the colon and ileum. However, in the colon of 3-year-old pigs, Firmicutes abundance was significantly reduced, whereas *Bacteroidota* and *Verrucomicrobiota* increases, resulting in a notable decrease in the *Firmicutes* to *Bacteroidota* (F/B) ratio. The rise of *Bacteroidota* indicates an enhanced capacity to degrade complex plant polysaccharides, suggesting that Tibetan pigs develop a greater adaptability to the plateau environment as they age [11]. At the species level, the abundance of fiber-degrading bacteria (*Prevotellaceae UCG 003*, uncultured *Ruminococcaceae*) and *Lactobacillus* (including *L. amylovorus* and *L. porci*) significantly increased in the colon of 3-year-old pigs. *Prevotella* and *Ruminococcus* are recognized as fiber-degrading bacteria that produce short-chain fatty acids, supporting colonic epithelial energy supply and barrier function [12]. *Lactobacillus* is a well-recognized probiotic that has been shown to inhibit pathogen colonization and plays a pivotal role in inhibiting pathogen colonization and sustaining intestinal homeostasis [13]. Notably, *Lactobacillus amylovorus* has been shown to enhance intestinal epithelial barrier function, and its increased abundance in older pigs further suggests improved barrier integrity. In the ileum of 3-year-old pigs, the abundance of certain commensal bacteria that may act as opportunistic pathogens (*Clostridium celatum*, *Romboutsia timonensis*, *Terrisporobacter glycolicus*) significantly decreased, whereas *Clostridium disporicum* (a butyrate-producing species) increased [14,15]. These changes point to a healthier and more stable gut microecosystem in 3-year-old Tibetan pigs.

Gut microbial metabolic activity is closely related to host age. Studies indicate that aging in animals is associated with a decline in microbial metabolic activity, down-regulation of host nucleotide metabolism, and an increase in systemic inflammation, suggesting that the deterioration of microbe-host metabolic interaction is a hallmark of aging [16]. In Neijiang pigs, age-related gut microbiota succession is accompanied by enhanced microbial functions such as ABC transporters and oxidative phosphorylation [17]. In the present study, colonic differential metabolites were primarily enriched in unsaturated fatty acid biosynthesis, tryptophan metabolism, and TRP channel pathways. Notably, metabolites such as DHA and arachidonic acid that were present at higher levels serve as key precursors for inflammatory mediators, suggesting that colonic mucosa may undergo age-related immune regulatory changes. In the ileum, metabolic alterations involved histidine metabolism, bile secretion, and ABC transporter pathways, highlighting its role in bile acid circulation and transmembrane transport. The increased abundance of *Lactobacillus amylovorus* and butyrate-producing *Clostridium* species in the colon of 3-year-old pigs, together with higher unsaturated fatty acids (docosahexaenoic and arachidonic acids), points to a coordinated shift toward an anti-inflammatory luminal environment. These pathway-specific metabolic shifts provide a biochemical basis for intestinal homeostasis in Tibetan pigs under high-altitude stress.

In the correlation analysis of 3-year-old Tibetan pig ileal microorganisms and genes, *UGT2B31* emerged as a up-regulated gene enriched in pathways related to bile secretion, retinol metabolism, and cytochrome P450-mediated xenobiotic metabolism, suggesting a role in host detoxification and metabolic regulation [18]. Spearman correlation analysis revealed a significant positive correlation between *UGT2B31* expression and the abundance of several bacterial genera, including *Lactobacillus equicursoris*, unclassified *Lachnospiraceae*, *Subdoligranulum*, and *Olsenella* suggesting a potential association with a gut microenvironment conducive to colonization by these bacteria. This association highlights a possible link between host metabolic maturation and microbial ecology in the plateau environment.

CCL28, a chemokine secreted by epithelial cells, recruits IgA+ plasma cells to the intestinal mucosa by binding to the CCR10 receptor. It serves as a crucial link between the epithelial barrier and adaptive immunity [19]. In this study, CCL28 was significantly upregulated and positively correlated with the butyrate-producing *bacterium* [*Eubacterium*] coprostanoligenes group. This gene was enriched in three immune-related pathways, suggesting its involvement in mucosal homeostasis and host defense. Concurrently, the abundance of the *Eubacterium coprostanoligenes* group was significantly positively correlated with the expression levels of upregulated genes such as *RBP2* (which mediates vitamin digestion and absorption) and *CACT* (which participates in fat digestion and absorption). This suggests a possible link between this bacterial group and host nutrient absorption pathways Furthermore, the increase in the abundance of *Lactobacillus porci* was positively correlated with the expression of the fatty acid transporter *FATP4*, suggesting an association between microbiota composition and host lipid metabolism. The up-regulation of genes involved in lipid metabolism (*FATP4*, *CACT*) and bile secretion (*UGT2B31*) is consistent with the observed increase in unsaturated fatty acids and bile secretion-related metabolites, supporting a coordinated shift in lipid handling and detoxification capacity in older pigs. Together, these correlations suggest a potential sIgA-centered host-microbiota interaction in the ileum of 3-year-old Tibetan pigs, warranting further mechanistic investigation.

While this study provides a comprehensive multi-omics overview of age-related intestinal transformations in Tibetan pigs, certain limitations should be acknowledged. The small sample size limits the generalizability of the findings, despite the use of stringent statistical thresholds. Additionally, while we observed distinct differences between 1-year-old and 3-year-old Tibetan pigs, the current study design does not fully decouple the effects of chronological aging from the concurrent dietary transition (from artificial supplementation to semi-grazing). Future studies with larger cohorts and controlled feeding designs are needed to validate these findings and dissect the effects of age versus diet.

## 5. Conclusions

This study investigates the differences in gut microbiota, metabolites, transcriptomes, and intestinal tissue morphology between 1-year-old and 3-year-old Tibetan pigs. The results indicate that as age increases, the small intestinal crypt depth in Tibetan pigs significantly decreases, alongside a notable reduction in the number of goblet cells. Conversely, the villus height and epithelial length remain stable, collectively correlating with a significant increase in the villus-crypt ratio (V/C). Compared to 1-year-old Tibetan pigs, the gut microbial diversity and abundance of butyrate-producing bacteria and *Lactobacillus*, are significantly higher in 3-year-old Tibetan pigs. Metabolomic and transcriptomic analyses reveal that the differential metabolites of Tibetan pigs at varying age stages are enriched in pathways related to unsaturated fatty acid, tryptophan, and histidine metabolism. Meanwhile, differentially expressed genes are significantly enriched in pathways associated with immune inflammation, nutrient digestion and absorption, and cell signaling. Correlational patterns suggest that in 3-year-old Tibetan pigs, the intestinal environment is associated with the colonization of butyrate-producing bacteria and *Lactobacillus* by upregulating key immune and metabolic pathways. Furthermore, these features are correlated with host barrier function and immune tolerance through mechanisms involving short-chain fatty acids and TLR signaling, pointing to a potential host-microbe symbiotic mechanism adapted to the plateau environment.

## Figures and Tables

**Table 1:** Alpha diversity index of each sample.

Sample	Feature_OTUs	ACE	Chao1	Simpson	Shannon	PD_Whole_Tree
C1-1	186	245.459	247.875	0.615	2.3658	16.0241
C1-2	170	206.4839	217.3	0.5675	2.1549	14.5555
C1-3	156	186.2279	193.0588	0.6018	2.2729	12.1041
C3-1	401	422.0955	421.5263	0.9304	5.5371	26.1471
C3-2	404	420.7644	427.8	0.9364	5.6613	27.5036
C3-3	375	385.18	392.1053	0.9347	5.5825	24.1498
I1-1	94	118.9989	121	0.7448	2.51	7.8312
I1-2	108	134.083	124.4348	0.7483	2.5202	9.2907
I1-3	116	142.7185	131.75	0.7524	2.5633	10.1984
I3-1	181	224.1637	232.75	0.4001	1.7526	16.2739
I3-2	191	231.4666	228.84	0.4459	1.921	16.1735
I3-3	200	290.8492	261.6	0.4662	1.9912	18.1447

## Data Availability

The data presented in this study are available on request from the corresponding author.

## Abbreviations

The following abbreviations are used in this manuscript:

CACT	Carnitine-acylcarnitine translocase
CCL28	C-C motif chemokine ligand 28
DHA	Docosahexaenoic acid
FATP4	Fatty acid transport protein 4
LDA	Linear discriminant analysis
LPC	Lysophosphatidylcholine
LPG	Lysophosphatidylglycerol
PI3K-Akt	Phosphoinositide 3-kinase-protein kinase B
RBP2	Retinol-binding protein 2
UGT2B31	UDP glucuronosyltransferase family 2 member B31
